# Nomograms Based on X-Ray Radiomics for Predicting Pain Progression in Knee Osteoarthritis Using Data From the Foundation for the National Institutes of Health: Development and Validation Study

**DOI:** 10.2196/78338

**Published:** 2026-01-14

**Authors:** Yingwei Sun, Jing Liu, Chunbo Deng, Chengbao Peng, Shinong Pan, Xueyong Liu

**Affiliations:** 1 Department of Radiology Affiliated Hospital of Liaoning University of Traditional Chinese Medicine Shenyang China; 2 Platform Engineering Research Center Neusoft Research Institute of Healthcare Technology Shenyang China; 3 Department of Sports and Trauma Central Hospital Affiliated to Shenyang Medical College Shenyang China; 4 Department of Radiology Shengjing Hospital of China Medical University Shenyang China; 5 Department of Rehabilitation Shengjing Hospital of China Medical University Shenyang China

**Keywords:** radiomics, osteoarthritis, knee, nomogram, artificial intelligence

## Abstract

**Background:**

Knee osteoarthritis (KOA) is one of the most prevalent chronic musculoskeletal disorders among the older adult population. Screening populations at risk of rapid progression of osteoarthritis and implementing appropriate early intervention strategies is advantageous for the treatment and prognosis of affected patients.

**Objective:**

This study aimed to construct and validate a nomogram model based on x-ray radiomics to effectively identify individuals experiencing progression of KOA pain.

**Methods:**

The Foundation for the National Institutes of Health Biomarkers Consortium included a total of 600 participants who were classified as pain progressors (n=297, 49.5%) and non–pain progressors (n=303, 50.5%) according to an increase in the Western Ontario and McMaster Universities Osteoarthritis Index pain score of ≥9 points (on a scale from 0 to 100) during the follow-up period of 24 to 48 months. X-rays that lacked defined spacing in the DICOM image were excluded. Fully automatic selection of subchondral bone regions on the inner and outer edges of the tibia and femur as regions of interest and extraction of radiomics features for different combinations of regions of interest were conducted. Least absolute shrinkage and selection operator regression was used to select features and generate a radiomics score using Shapley additive explanations for interpretability. The radiomics score, along with clinical indicators, was incorporated into nomograms using a multivariable logistic regression model. The subgroup analysis focused solely on the progression of pain and cases with no progression at all. The receiver operating characteristic curve, along with calibration and decision curves, was used to assess the discriminative performance.

**Results:**

A total of 450 participants were included in the study. Shapley additive explanations analysis identified Wavelet-HH_gldm_HighGrayLevelEmphasis as the primary radiomics feature. Nomogram 1 and nomogram 2 for predicting KOA pain progression achieved area under the curve values of 0.766 and 0.753, respectively, with mean absolute errors of 0.012 and 0.008, respectively, in the calibration curves. Decision curve analysis showed a positive net benefit across a range of threshold probabilities. In subgroup analyses, nomogram 3 and nomogram 4 yielded areas under the curve of 0.795 and 0.740, respectively.

**Conclusions:**

The nomograms based on x-ray radiomics demonstrated excellent predictive capability and accuracy in forecasting the progression of KOA pain.

## Introduction

Osteoarthritis (OA) is one of the most common joint disorders affecting the older adult population. With the aging of the population, its incidence has been increasing year by year, imposing a significant burden on both individuals and society [1]. Knee OA (KOA) has the highest incidence among all types of OA. The etiology of KOA is multifactorial, and its pathogenesis remains unclear. KOA can be categorized into various clinical phenotypes and molecular subtypes, including metabolic, inflammatory, mechanical, and genetic [2]. The current treatment guidelines are not suitable for the early diagnosis of KOA and do not distinguish patients who may experience rapid progression [3]. Identifying patients at high risk of rapid progression of KOA will assist in patient education and in the prioritization and allocation of health care resources [4].

The subchondral bone is essential for knee joint structure. Its degeneration disrupts the homeostasis of the intra-articular environment, potentially triggering cartilage degeneration and injury. Furthermore, it is associated with the incidence and progression of OA [5,6]. X-ray imaging continues to be the most prevalent, convenient, and cost-effective modality for evaluating KOA. However, it can only provide a rough estimate of disease severity without quantitative analysis or predicting disease progression [7]. With the application of artificial intelligence in KOA, x-rays can now predict the progression of KOA [3]. Leung et al [8] developed a deep learning predictive model to assess the risk of progression in KOA using knee x-ray images. The findings of their study indicated that this deep learning model surpassed traditional binary outcome models, which are based on standard scoring systems, in predicting the likelihood of total knee arthroplasty among patients with OA [8]. Although deep learning technology is regarded as the most sophisticated image classification technique currently available, its inherent lack of interpretability poses challenges for users in comprehending the model’s decision-making outcomes [9].

Radiomics is a novel approach that harnesses advanced image analysis tools and the rapid development of medical imaging data. By integrating artificial intelligence, it enables high-throughput extraction of information from standard medical images and applies the extracted data to clinical decision support systems to enhance the accuracy of diagnosis, prognosis, and prediction [10]. Radiomics has been gradually applied in the field of KOA. Current research primarily focuses on using radiomics to diagnose KOA and predict its radiological progression [11-14]. However, studies on radiomics for predicting the pain progression of KOA are still relatively scarce. A recent study that used magnetic resonance imaging (MRI) radiomics to predict pain progression in KOA demonstrated that the constructed radiomics model achieved an area under the receiver operating characteristic curve (AUC) of 0.79 to 0.86 for KOA pain progression prediction [15]. Nevertheless, the study incorporated 200 radiomics parameters and included omics data from 12- and 24-month follow-ups, resulting in high model complexity and limited practical applicability.

Therefore, this study aimed to develop a nomogram model integrating x-ray subchondral radiomics features and clinical characteristics to predict pain progression in KOA. The Shapley additive explanations (SHAP) method was incorporated to offer a comprehensive explanation and intuitive visualization of the model’s predictive mechanisms. This approach allows for the identification of high-risk individuals with rapid pain progression among patients with KOA using cost-effective detection methods, thereby providing clinicians with evidence-based guidance for early intervention strategies.

## Methods

### Participants

**Figure 1:**
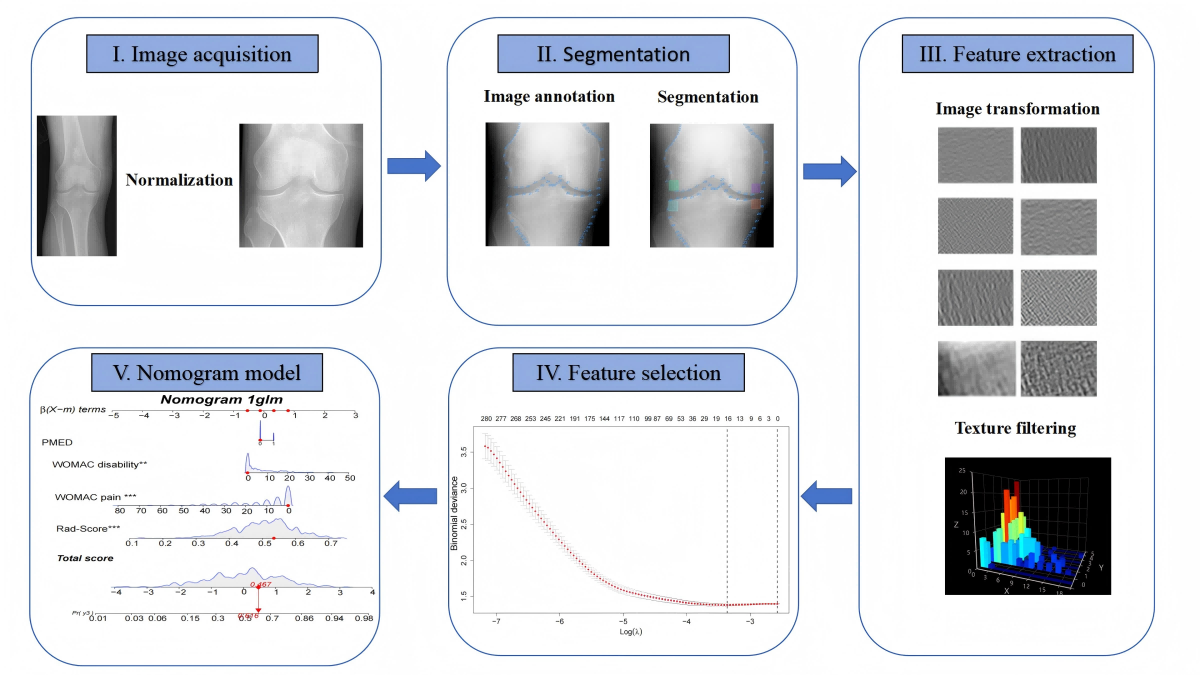
Flowchart of the study. The x-ray images of the knee joint were normalized, and the regions of interest were automatically identified and selected. Subsequently, imaging omics parameters were extracted, followed by a screening process for feature parameters. Imaging omics labels were then constructed to evaluate clinical features, ultimately leading to the generation of a nomogram and an assessment of its performance.

This study involved participants from the Foundation for the National Institutes of Health (FNIH) Osteoarthritis Biomarkers Project. The design of this research is detailed in previously published literature [16,17]. In brief, the FNIH Osteoarthritis Biomarkers Project includes 600 participants with KOA of Kellgren-Lawrence grades 1 to 3. According to the results of the follow-up, the 600 participants at baseline were categorized into four distinct subgroups: (1) participants exhibiting both radiological and pain progression (n=194, 32.3%), (2) participants with only radiological progression (n=103, 17.2%), (3) participants experiencing only pain progression (n=103, 17.2%), and (4) participants who exhibited neither radiological nor pain progression (n=200, 33.3%). Radiological progression was defined as a loss of minimum joint space width of ≥0.7 mm in the medial femorotibial compartment between baseline and follow-up at 24, 36, or 48 months. Pain progression was characterized by a sustained increase in the Western Ontario and McMaster Universities Osteoarthritis Index (WOMAC) pain subscale (≥9 on a scale from 0 to 100) based on the minimum clinically important difference during the same time frame. In the Digital Imaging and Communications in Medicine (DICOM) standard, pixel spacing is a critical factor for converting image pixels into physical dimensions. Spacing ensures the reliability and reproducibility of medical images in diagnosis, research, and treatment planning [18]. However, some datasets lack or contain erroneous spacing information. They use default values (1.0, 1.0), which result in calculated knee dimensions significantly exceeding normal human anatomical ranges. Such data can be defined as “DICOM data with invalid spacing information.” In the standardization process for the Osteoarthritis Initiative (OAI) knee x-ray images, cases with unspecified pixel spacing were excluded. As the pixel spacing of 0.2 mm was predominant in the dataset, all retained images were calibrated to this value to ensure consistent spatial scaling [19]. The specific process of normalization is described in Multimedia Appendix 1 [20-22]. Groups 1 and 3, which showed pain progression, were combined as the case group, whereas groups 2 and 4, with no pain progression, served as the control group for analysis. Additionally, we conducted subgroup analyses on group 3 (with only pain progression) and group 4 (without both pain and radiological progression). The study flow diagram is shown in Figure 1.

### Segmentation and Region of Interest Annotation

**Figure 2:**
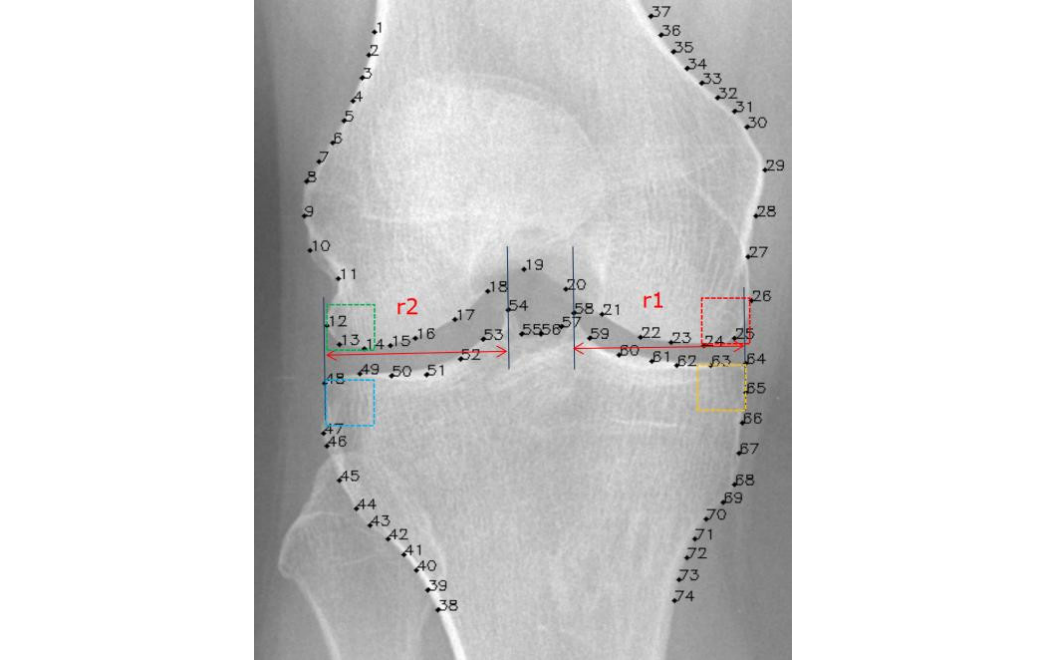
Schematic diagram of the automated region of interest segmentation process.

BoneFinder (The University of Manchester) is a fully automated software tool designed to outline and segment skeletal structures from 2D radiographs by placing a series of points along bone contours or at key anatomical landmarks. In brief, BoneFinder uses a random forest voting mechanism to precisely determine the optimal placement of model feature points, enabling robust and accurate shape model matching. The system can accurately identify and segment bones not previously present in new images. This method demonstrates exceptional robustness and precision across various skeletal applications, with its generated point data being widely applicable to morphometric analyses, including the construction of statistical morphological models and automatic derivation of standard geometric measurement parameters [23], resulting in 74 markers per image (Figure 2). The fully automatic BoneFinder knee module achieves a mean point-to-curve error of less than 1.0 mm for 99% of 500 images (ie, in 99% of images, the error is less than 1.0 mm) without considering knee size [24].

Four vertical lines are constructed at the marked points 64, 58, 54, and 48 on the knee joint x-ray image, as identified by BoneFinder. These lines are sequentially designated as line 1, line 2, line 3, and line 4. The distance between line 1 and line 2 is denoted as R1, whereas the distance between line 3 and line 4 is referred to as R2. With point 64 designated as the vertex and a side length of R1/4, a square encompassing the medial tibia was defined as the region of interest (ROI). With marker point 64 designated as the vertex and a quarter of R1 as the side length, a square encompassing the medial tibia was defined as the ROI. First, using marker point 48 as the vertex and a quarter of R2 as the side length, a square area on the lateral tibia was selected to define ROI. Second, the selection process for the femur ROI differs from that of the tibia ROI. ROI 3 is defined with the line intersecting marker 24 as its lower boundary, while the line intersecting marker point 26 serves as the side boundary of this ROI. ROI 4 of the lateral femur uses the line indicated at point 14 as the lower boundary of the ROI and the column line marked at point 12 as its vertical boundary. Then, squares with side lengths of a quarter of R1 and a quarter of R2 are constructed. To assess the repeatability of ROI delineation, a total of 40 cases were randomly selected from the collected dataset. Two senior musculoskeletal radiologists (YWS and SNP) conducted manual segmentation of ROIs. Dice similarity coefficients were used to assess manual and automated segmentation agreement. Image annotation was performed using Neusoft Darts [25].

### Radiomics Feature Extraction

The extraction of radiomics features was based on Neusoft Discovery [25]. The features that were extracted can be categorized into 3 distinct groups. First-order features were derived from the gray values of images, reflecting the distribution and heterogeneity of intensity within the ROI, typically including maximum, minimum, mean, median, range, variance, kurtosis, skewness, and entropy. The second-order features refer to the extracted texture characteristics, which include texture feature values derived from the gray-level co-occurrence matrix, gray-level run-length matrix, gray-level connected region size matrix, neighborhood difference matrix, and gray-level correlation matrix. The texture features can quantify the distribution of textures within the ROI that are difficult to perceive visually. Third, features were extracted after log transformation, first-level wavelet decomposition, and gradient transformation were applied to the ROI. All features were normalized using the following formula: *Z*=(*x*–μ)/σ.

### Radiomics Feature Selection and Radiomics Signature Construction

The Spearman correlation was used to remove the redundant features. The feature subset was optimized using the least absolute shrinkage and selection operator (LASSO) method in logistic regression. A 10-fold cross-validation approach was used to identify the optimal model that minimizes the mean squared error, and the correlation coefficients of the features selected by the best LASSO model were calculated. According to the coefficients derived from LASSO regression, a weighted calculation was performed to obtain the radiomics score (rad-score). Radiomics parameters from 4 ROIs (ROIs 1-4) were extracted individually, and rad-score 1 was developed following LASSO screening of the nonzero parameters. The radiomics parameters were applied to ROI 1 and ROI 3, leading to the development of rad-score 2. In subgroup analysis, rad-score 3, obtained from extracted and screened nonzero parameters from ROIs 1 to 4, and rad-score 4, obtained from extracted and screened nonzero parameters from ROI 1 and ROI 3, predicted pain progression only and no progression at all, respectively. SHAP analysis was used to mitigate the “black-box” effect of machine learning, enabling feature prioritization and interpretability of individual radiomics features on the rad-score. Feature maps were used to visualize the results of the rad-score.

### Development and Evaluation of Nomograms

The analysis used stepwise backward multivariable logistic regression to identify clinical risk factors at baseline and construct nomograms. The Akaike information criterion likelihood ratio test was used as the stopping rule for the backward stepwise logistic regression analysis. Clinical information from patients at baseline included age, sex, history of injury, BMI, the minimum joint space width of the medial tibial-femoral compartment, Kellgren-Lawrence grade, WOMAC pain score and WOMAC disability score, medicine for pain in the previous 12 months, and race. The nomograms were developed based on the prediction probability value of the optimal radiomics models and clinical independent risk factors. The discriminative ability of the nomogram was evaluated using the AUC and accuracy. Calibration of the nomogram was performed through calibration curves, with optimal calibration indicated by alignment with the diagonal line. Internal validation was conducted using a bootstrap method involving 1000 resampling iterations, and the mean absolute error was used to assess model calibration. Additionally, decision curve analysis (DCA) quantified net benefits at various threshold probabilities to evaluate the clinical effectiveness of the nomograms.

### Statistical Analysis

The comparison of categorical variables was conducted using the Pearson chi-square test. Continuous variables were expressed as mean and SD and analyzed using either the 2-tailed *t* test or Mann-Whitney *U* test. The normality of continuous data was assessed using the Kolmogorov-Smirnov test; measurements that followed a normal distribution were subjected to the *t* test, whereas those that did not conform to a normal distribution underwent the Mann-Whitney *U* test. The DeLong test was used for comparing 2 models. *P* values were calculated using a 2-tailed *z* test, with *P*<.05 considered statistically significant. All statistical analyses were performed using the EmpowerStats software (X&Y Solutions, Inc) and the R software (version 4.2.2; R Foundation for Statistical Computing).

### Ethical Considerations

This study was conducted in accordance with the Declaration of Helsinki (as revised in 2013). The Institutional Review Board of the University of California, San Francisco (approval 10-00532), and the 4 clinical centers of the OAI project recognized the study as HIPAA (Health Insurance Portability and Accountability Act) compliant. All participants provided written informed consent. Patient-identifiable information (including names and hospital ID numbers) was carefully removed from the x-ray images and reports before analysis. Due to the retrospective design of this study, participants received no compensation.

## Results

### Baseline Clinical Characteristics

Due to the lack of spacing in DICOM images, a total of 450 participants were ultimately included in the study, with 227 (50.4%) and 223 (49.6%) in the control and case groups, respectively. The baseline characteristics of participants in both the case and control groups were frequency matched for factors including age, BMI, WOMAC functional scores, Kellgren-Lawrence grade, pain medication history, and race. Notably, a statistically significant difference was observed in WOMAC pain scores (*P=*.04) and medication history (*P*=.02) between the 2 groups (Table 1). In subgroup analysis, 18% (n=81) of the individuals demonstrated only pain progression during the follow-up period, whereas 32.9% (n=148) of the individuals exhibited neither pain progression nor radiographic deterioration. The baseline characteristics of participants across both groups were well matched, with no statistically significant differences observed (Table S1 in Multimedia Appendix 2).

**Table 1:** Baseline characteristics of the study population.

Characteristic				Control group (n=223)		Case group (n=227)		*P* value	
**Demographics**									
	Age (years), mean (SD)			62.14 (8.91)		61.48 (8.79)		.38	
	Female sex, n (%)			131 (58.7)		136 (59.9)		.80	
	**Race, n (%)**								.44
		Asian	3 (1.3)		2 (0.9)				
		Black or African American	21 (9.4)		32 (14.1)				
		White	196 (87.9)		191 (84.1)				
		Other non-White race	3 (1.3)		2 (0.9)				
**Clinical measures**									
	BMI (kg/m^2^), mean (SD)			30.36 (4.85)		30.50 (4.71)		.75	
	WOMAC^a^ pain score (0-20), mean (SD)			2.70 (3.40)		1.87 (2.47)		.04	
	WOMAC function score (0-68), mean (SD)			8.15 (10.98)		8.13 (9.18)		.15	
	History of knee injury, n (%)			77 (34.5)		83 (36.6)		.65	
	Use of pain medication, n (%)			49 (22.0)		73 (32.2)		*.*02	
**Radiographic measures**									
	**Kellgren-Lawrence grade, n (%)**								.70
		1	29 (13.0)		29 (12.8)				
		2	112 (50.2)		106 (46.7)				
		3	82 (36.8)		92 (40.5)				
	Minimum medial joint space width (mm), mean (SD)			3.77 (1.10)		3.80 (1.32)		.87	

^a^WOMAC: Western Ontario and McMaster Universities Osteoarthritis Index.

### Segmentation and Feature Extraction

The automated segmentation dataset was used for radiomics feature extraction as the Dice similarity coefficients between automatic and manual segmentation were excellent (0.800; Table S2 in Multimedia Appendix 2). We used 10-fold cross-validation to identify the optimal tuning parameter (alpha). In total, 16 nonzero coefficient features were derived from the optimal tuning parameters in rad-score 1. Rad-score 2 comprised 12 nonzero coefficient features. In subgroup analysis, rad-score 3 included 11 nonzero feature parameters, whereas rad-score 4 consisted of 3 nonzero features (Tables S3-S6 in Multimedia Appendix 2 and Figures S1-S4 in Multimedia Appendix 2). The correlation among features was analyzed using Spearman correlation analysis, and heat maps are presented in Figures S1 to S4 in Multimedia Appendix 2.

### Development and Evaluation of Radiomics Signatures

**Figure 3:**
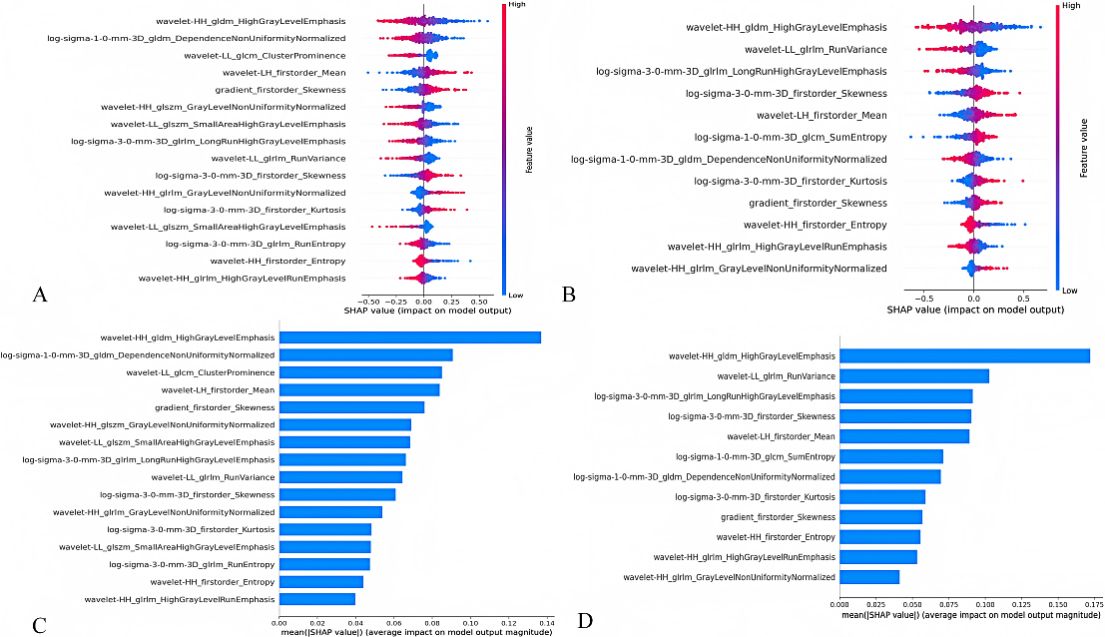
Shapley additive explanations (SHAP) summary plots of the radiomics score. The plot illustrates the feature relevance and attributions of the model’s predictive performance: (A) rad-score 1 and (B) rad-score 2. The bar chart illustrates the impact of various imaging features on the rad-score: (C) rad-score 1 and (D) rad-score 2.

**Figure 4:**
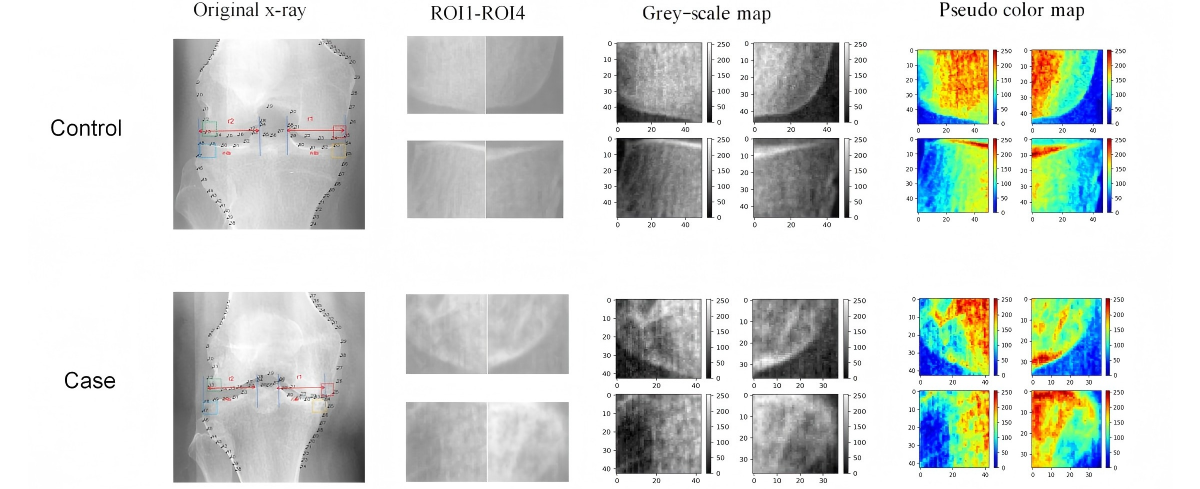
Visualization of radiomics signatures. The case group participant demonstrated higher region of interest heterogeneity than the control group participant, with a higher score.

Table 2 shows the diagnostic proficiency of 4 radiomics signatures. The AUC value for rad-score 1 was 0.728 (95% CI 0.681-0.774), the AUC value for rad-score 2 was 0.716 (95% CI 0.635-0.736), the AUC value for rad-score 3 was 0.775 (95% CI 0.714-0.837), and the AUC value for rad-score 4 was 0.679 (95% CI 0.604-0.755; Table 2). The SHAP plot in Figure 3 visually illustrates how radiomics features influenced model predictions in terms of magnitude and direction. Features are sorted vertically by global importance. Each point represents the SHAP value of each feature for a specific patient plotted horizontally and stacked vertically to show density distribution. The color of the point reflects the feature value, from low (blue) to high (red; Figures 3A and C). The bar chart illustrates the impact of various imaging features on the rad-score. Wavelet-HH_gldm_HighGrayLevelEmphasis showed significant effects on both rad-score 1 and rad-score 2 (Figures 3B and D). The SHAP values of each feature in rad-score 3 and rad-score 4 are shown in Figure S5 in Multimedia Appendix 2. Figure 4 shows representative participants with x-ray subchondral bone feature maps from 4 ROIs. The case group participant demonstrated higher ROI heterogeneity than the control group participant, with a higher score (0.58 vs 0.44).

**Table 2:** Diagnostic efficacy of the 4 radiomics scores.

Radiomics score	AUC^a^ (95% CI)	Accuracy	Sensitivity	Specificity
1	0.728 (0.681-0.774)	0.684	0.797	0.570
2	0.716 (0.635-0.736)	0.668	0.683	0.677
3	0.775 (0.714-0.837)	0.742	0.753	0.716
4	0.679 (0.604-0.755)	0.694	0.580	0.757

^a^AUC: area under the receiver operating characteristic curve.

### Construction and Testing of Nomograms

**Figure 5:**
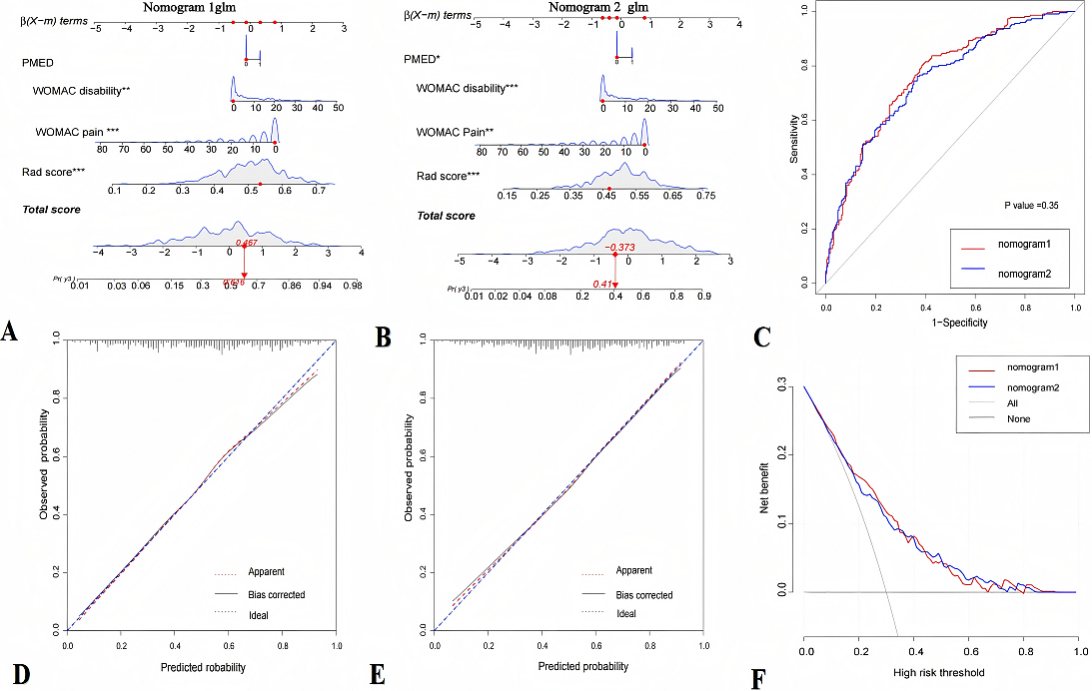
Development and validation of the nomogram to predict pain progression in knee osteoarthritis (A: nomogram 1; B: nomogram 2), the receiver operating characteristic curves of nomogram 1 and nomogram 2(C), the calibration curves of nomogram 1 and nomogram 2 (D and E, respectively), and decision curves for nomogram 1 and nomogram 2 (F). PMED: Use of pain medication.

Through multiple regression analysis, the WOMAC disability score, pain medication history, and WOMAC pain score were found to be risk factors for KOA pain progression. We constructed visual nomograms by integrating the score with selected clinical risk factors. The AUC of nomogram 1 was 0.766 (95% CI 0.722-0.809), and the AUC of nomogram 2 was 0.753 (95% CI 0.708-0.797). The comparison of AUC between the 2 models showed no statistically significant difference (DeLong test; *P*>.05; Figure 3). The calibration curve demonstrated that all cohorts were accurately aligned with the actual observations. The internal validation was conducted using the bootstrap method, with a resampling count set to 1000. The mean absolute errors of the calibration curves for the 2 models were found to be 0.012 and 0.008, indicating that the model demonstrated a high level of calibration accuracy (Figure 5 and Table 3). The DCA demonstrated that, if the threshold probability in the clinical decision was in the range of 10% to 70%, both nomogram 1 and nomogram 2 could yield more net benefits compared with either the treat-all-patients strategy or the treat-none strategy.

**Table 3:** Diagnostic efficacy of the 4 nomogram models.

Model	AUC^a^ (95% CI)	Accuracy	Sensitivity	Specificity	MAE^b^
Nomogram 1	0.766 (0.722-0.809)	0.709	0.692	0.726	0.012
Nomogram 2	0.753 (0.708-0.797)	0.693	0.648	0.740	0.008
Nomogram 3	0.795 (0.734-0.865)	0.760	0.691	0.797	0.011
Nomogram 4	0.740 (0.673-0.808)	0.686	0.740	0.695	0.013

^a^AUC: area under the receiver operating characteristic curve.

^b^MAE: mean absolute error.

### Subgroup Analyses

**Figure 6:**
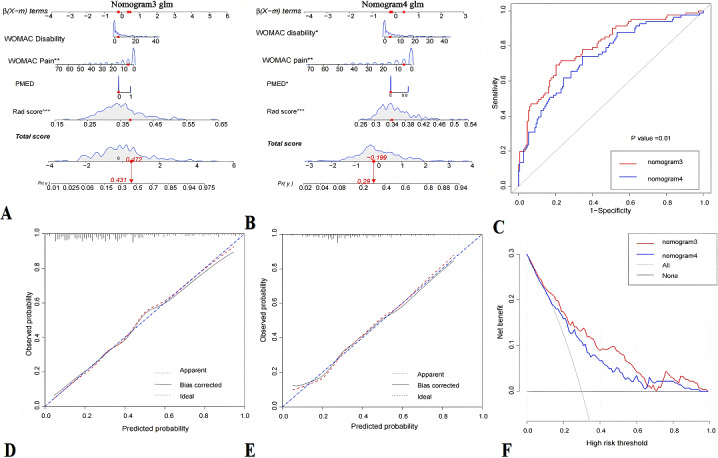
Development and validation of the nomogram to predict pain progression in knee osteoarthritis (A: nomogram 3; B: nomogram 4), the receiver operating characteristic curves of nomogram 3 and nomogram 4 (C), the calibration curves of nomogram 3 and nomogram 4 (D and E, respectively), and decision curves for nomogram 3 and nomogram 4 (F). PMED: Use of pain medication.

The AUC of nomogram 3 was 0.795 (95% CI 0.734-0.865), and the AUC of nomogram 4 was 0.740 (95% CI 0.673-0.808). The comparison of the AUC between the 2 models showed a statistically significant difference (DeLong test; *P*<.05). The calibration curve demonstrated a robust concordance between the probabilities predicted and the actual observed outcomes. The DCAs demonstrated that, if the threshold probability in the clinical decision was within the range of 10% to 65%, the curve corresponding to nomogram 3 was above nomogram 4, and the AUC that it formed with the “treat none” and “treat all” lines was relatively larger. The net benefit of nomogram 3 was superior to that of nomogram 4, making it the better model (Table 3 and Figure 6).

## Discussion

### Principal Findings

Our study developed a nomogram integrating x-ray radiomics features and clinical variables to predict pain progression in KOA. The resulting baseline-based, cost-effective nomogram demonstrated commendable accuracy and reproducibility in forecasting KOA pain progression, offering significant potential for guiding early intervention strategies in at-risk populations.

The application of advanced artificial intelligence to widely available and cost-effective x-ray imaging represents a feasible and promising approach for predicting KOA pain progression. Radiomics models can automatically classify KOA [26], and texture analysis of the subchondral bone, particularly in the tibia, has shown predictive value for radiographic KOA incidence [27,28]. Given the complex etiology of KOA and the frequent discrepancy between clinical symptoms and radiological severity, we focused on subchondral bone remodeling—a process linked to pain pathogenesis through molecules such as netrin-1 and PEG2, which promote sensory nerve innervation [29,30]. This rationale supported our use of subchondral bone radiomics for predicting symptomatic progression. Radiomics enables high-throughput extraction of quantitative features from medical images, providing data for clinical decision support systems [10]. The selection of key ROIs is critical as different image areas contain varying levels of information relevant to disease progression [31]. While the medial subchondral bone often shows a stronger correlation with OA [32] and the medial tibial margin is recognized as highly informative due to its role in absorbing uneven mechanical loads [33], the optimal ROI selection remains debated. Some deep learning studies for KOA pain progression have also concentrated on the medial joint space and osteophytes [8], whereas other radiomics research suggests that including the entire tibial subchondral bone may yield better outcomes than focusing solely on specific compartments [17].

In this study, we systematically evaluated the impact of ROI selection on model accuracy by constructing radiomics signatures both from 4 ROIs encompassing the entire knee joint and from the 2 medial ROIs alone. Our findings revealed that incorporating lateral ROIs did not improve the overall model AUC compared to using medial ROIs only. However, subgroup analysis indicated that models combining medial and lateral ROIs achieved significantly higher AUC values in specific contexts. The primary cause of this discrepancy may stem from disease heterogeneity. Notably, in the main analysis, both the pain progression group and the nonprogression group included participants with radiological progression. Pathological changes in the lateral subchondral bone may accelerate the radiological progression of the knee joint [34-36]. Therefore, the role of radiomics features in lateral ROIs during pain progression was likely diluted or masked by features from radiologically progressive participants in the control group.

When compared to prior studies, our model demonstrated competitive predictive performance. For instance, Kraus et al [16] applied 3 texture parameters to predict KOA pain progression, achieving AUCs ranging from 0.603 to 0.649. Our rad-score yielded AUCs between 0.679 and 0.775, representing a substantial improvement. This aligns with the understanding that multivariable models integrating multiple risk factors generally provide more reliable estimates than those based on single parameters [37]. The superior performance of our baseline-based model is particularly notable compared to previously reported predictive capabilities relying solely on baseline biomarkers [38]. A recent study using MRI-based radiomics to predict KOA pain progression reported high predictive accuracy, with AUC values ranging from 0.79 to 0.86 [15]. This performance is commendable. However, it is important to note that their model incorporated a substantially larger set of 200 radiomics parameters and, crucially, relied on omics data extracted from 12- and 24-month follow-up MRI scans. While this approach yields high accuracy, the model’s complexity and its dependence on longitudinal imaging data significantly limit its practicality and scalability in routine clinical settings. In contrast, our study deliberately pursued a more streamlined and clinically translatable strategy. By using only baseline x-ray images and constructing a parsimonious model using key radiomics features and clinical variables, our baseline-based model eliminates the need for costly follow-up MRI scans and complex feature sets, making it more feasible for immediate clinical application and potential widespread adoption.

Research using deep learning methods to predict KOA pain progression remains scarce. A study using OAI data demonstrated that deep learning models achieved an AUC of 0.77 for predicting KOA pain progression, significantly higher than the 0.692 of traditional models [39]. By integrating demographic, clinical, and imaging risk factors with a deep learning model incorporating baseline knee x-rays, the highest diagnostic efficacy (AUC=0.807) was achieved in predicting pain progression. However, the original study failed to provide specific feature information. In contrast, our study visually presented each feature parameter’s impact on the rad-score using Shapley summaries. Wavelet-HH_gldm_HighGrayLevelEmphasis, a gray-level correlation matrix feature derived from high-frequency (HH) subbands of wavelet-transformed images, reflects the concentration of high–gray value regions and emphasizes texture variations in these areas. By quantifying texture changes in knee images, this feature effectively captures tissue degeneration differences caused by pain progression, indirectly reflecting pathological changes and potentially enabling prediction of knee pain progression.

The nomogram was selected for its ability to simplify complex statistical models into user-friendly graphical interfaces that generate individualized risk estimates [40]. While nomograms have been applied in OA contexts such as predicting outcomes after total knee replacement [41,42], their application specifically for predicting KOA pain progression represents a novel contribution. Our nomogram uniquely integrates radiomics signatures with key clinical variables: baseline WOMAC pain scores, WOMAC disability scores, and analgesic use. Interestingly, the baseline WOMAC pain score served as a negative predictor in our model, potentially reflecting the development of tolerance to chronic pain in patients with KOA [43]. The inclusion of WOMAC disability scores and analgesic use is supported by their established roles as risk factors in KOA phenotypic and prognostic models [44,45].

### Limitations

This study has several limitations. First, the relatively small sample size may affect statistical power and generalizability. Second, the use of data from previous nested case-control studies could introduce selection bias. Third, our analysis relied solely on internal validation because it was difficult to find an equivalent external cohort. Although the data were sourced from the well-established FNIH Osteoarthritis Biomarkers Project, external validation using independent datasets, such as those from the PROGRESS OA study (phase 2 of the FNIH Biomarkers consortium), was not feasible as these data have not been publicly released [46]. To our knowledge, no other studies have explored the specific pain progression patterns defined by the FNIH [15], and other studies using FNIH data have similarly been unable to perform external validation.

### Conclusions

A nomogram based on baseline x-ray radiomics signatures can effectively predict the progression of pain in KOA. This has significant implications for supporting health care professionals in tailoring treatment plans for individuals experiencing rapid pain progression, as well as in reducing health care costs, and these findings need further validation in future trials.

## Appendix

Multimedia Appendix 1 Size normalization and pixel normalization of the x-ray.

Multimedia Appendix 2 Baseline characteristics of the subgroup population, radiomic characteristics, and Dice and Shapley additive explanations summary plots of the radiomic score.

## Abbreviations

AUC: area under the receiver operating characteristic curve
DCA: decision curve analysis
DICOM: Digital Imaging and Communications in Medicine
FNIH: Foundation for the National Institutes of Health
HIPAA: Health Insurance Portability and Accountability Act
KOA: knee osteoarthritis
LASSO: least absolute shrinkage and selection operator
MRI: magnetic resonance imaging
OA: osteoarthritis
OAI: Osteoarthritis Initiative
rad-score: radiomics score
ROI: region of interest
SHAP: Shapley additive explanations
WOMAC: Western Ontario and McMaster Universities Osteoarthritis Index

## Data Availability

The datasets generated or analyzed during this study are available in the Osteoarthritis Initiative repository [47].
