# Supplementary material for: Nomograms Based on X-Ray Radiomics for Predicting Pain Progression in Knee Osteoarthritis Using Data From the Foundation for the National Institutes of Health: Development and Validation Study
Source: JMIR Med Inform. 2026 Jan 14;14:e78338. doi: 10.2196/78338 (PMC12853086; doi:10.2196/78338)
Supplement: Multimedia Appendix 1 [file medinform_v14i1e78338_app1.docx]

## **Multimedia Appendix 1.**

1) Pixel normalization

Due to different devices or parameter configurations, the pixel intensity values of similar positions on different X-ray films differed significantly (Figure 1). The maximum and minimum value normalization method was applied, that is, 98% and 2% of the maximum value of the X-ray pixel intensity of the entire knee joint were taken as the two reference values a1 and a2, and the ratio of each pixel intensity value i to these two reference values, namely (i-a2)/(a1-a2), was calculated. The data of the whole image was converted to a value between 0 and 1, so that the pixel intensity values at the same position of different images were relatively close, which is conducive to the processing of the depth learning neural network (Figure 2).

The maximum and minimum value normalization method is used to select a group of maximum and minimum values for each point on the H * W image.

$$I_{i}=\left\{ \begin{aligned} I_{min}, I_{i}<I_{min} \\ I_{i},I_{min}\leq I_{i}<I_{max} \\ I_{max},I_{i}\geq I_{max} \end{aligned} \right.$$

$$I_{i}^{*}=\frac{I_{i}-I_{min}}{I_{max}-I_{min}}$$

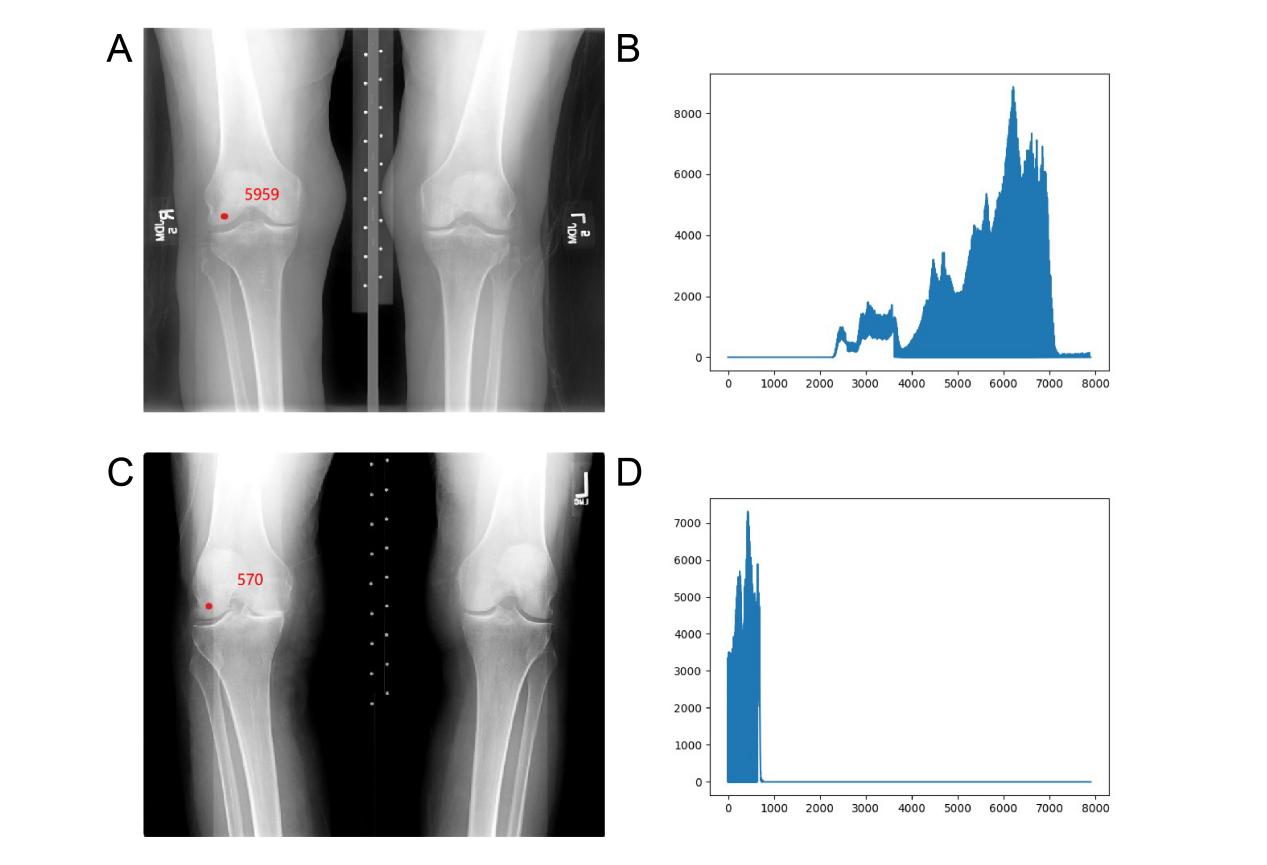


Figure 1. Histogram of knee joint pixel intensity. Figs. A and C: Pixel intensity value of a point on the lateral femoral condyle of the knee joint X-ray; Figs. B and D: intensity distribution histograms of different X-rays (0 value has been removed). The abscissa represents the intensity value, and the ordinate represents the number of different intensity values. Figs. A and C show that the pixel intensity of the same site in different X-rays is significantly different. The intensity distribution histogram shows that the intensity distribution on the two X-ray films is uneven.


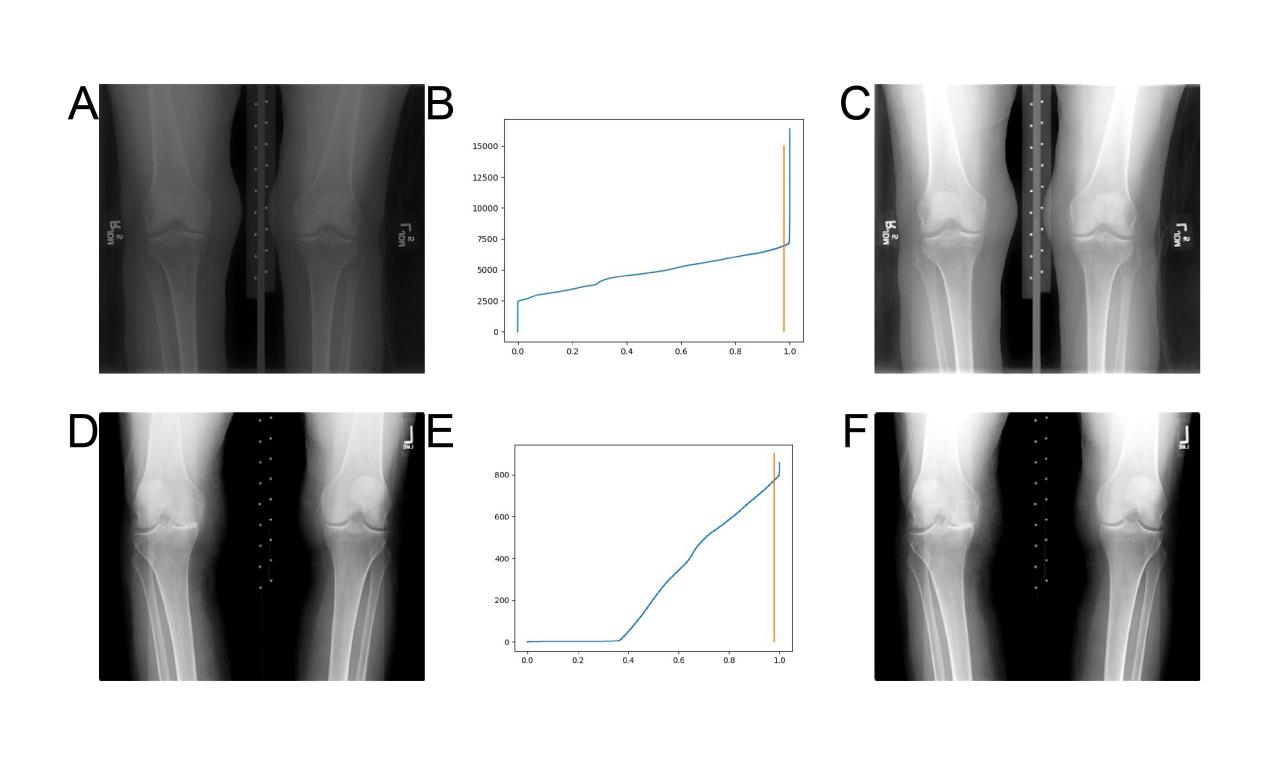


Figure 2. X-ray image is normalized by pixels. Figs. A and D are the images before and after normalization using the maximum and minimum intensity values. Due to the influence of some luminance outliers, the normalization effect is not good. Figs. C and F are the images before and after normalization, using 98% and 2% median intensity values as the maximum and minimum values. Figs. B and E are the distribution maps of pixel intensity sorting, and the abscissa is the percentage of data, where the orange line is the median value of 98%. Using the maximum value of 98% as the normalization parameter can well eliminate the brightness outliers.

2) Size normalization

We read the "pixel spacing" of the image from the Osteoarthritis Initiative database dicom file (see Figure 3). The data with a pixel spacing of 1.0 is the default value adopted due to missing parameters. As shown in Figure 3, most pixel spacing is ≤ 0.2. Adjust the pixel spacing uniformly to 0.2, including the high and wide resolution range of the image (Table S1). Therefore, in the subsequent operations of this study, the images were uniformly adjusted to a resolution of 2240 * 2240. The excess parts were symmetrically cropped on both sides, and the insufficient parts were filled with zeros on one side.

In this study, we adjusted the image spacing to 0.2 in the image preprocessing process, where the spacing represents the physical size of the image [1]. By adjusting the spacing to achieve normalization of the image size, we projected the true knee joint gap and improved the accuracy of grading. Few studies have focused on the "PixelSpacing" label in the image file in the image preprocessing process[2]. The OAI database image acquisition method is divided into Computational Radiography (CR), which refers to the digital image collected by the device using an X-ray sensitive plate, which is then read by the processor, and the digital X-ray flms (DR) [3]. Consistent with early research, we normalize pixel intensity during the preprocessing process, reduce the impact of noise, and correct the contrast of overexposed and unexposed images [2,4]. The data of pixel spacing 1.0 is the default value due to missing parameters. The pixel spacing was uniformly adjusted to 0.2 to incorporate the high and wide resolution range of the image (Table 1). Therefore, in the subsequent operation of this study, the images were uniformly adjusted to the resolution of 2240 x 2240. The excess part was cut symmetrically on both sides, and the insufficient part was made up with zero on one side.

Previous studies on applying OAI data for deep learning used the image resolution of 224 × 224-512 × 512 pixels. A recent study on the CAD of rheumatoid arthritis based on deep learning compared the diagnostic accuracy of different resolutions and found that when the resolution was increased to 608 × 608, the multi-classification accuracy was higher than that of 320 × 320. The image file of OAI shows that the image resolution was approximately 700 × 700. We adjusted the image resolution to 640 × 640 to ensure the accuracy of the model. A high-resolution image represents more details, while a lower-resolution image causes a loss of considerable information, resulting in lower accuracy.


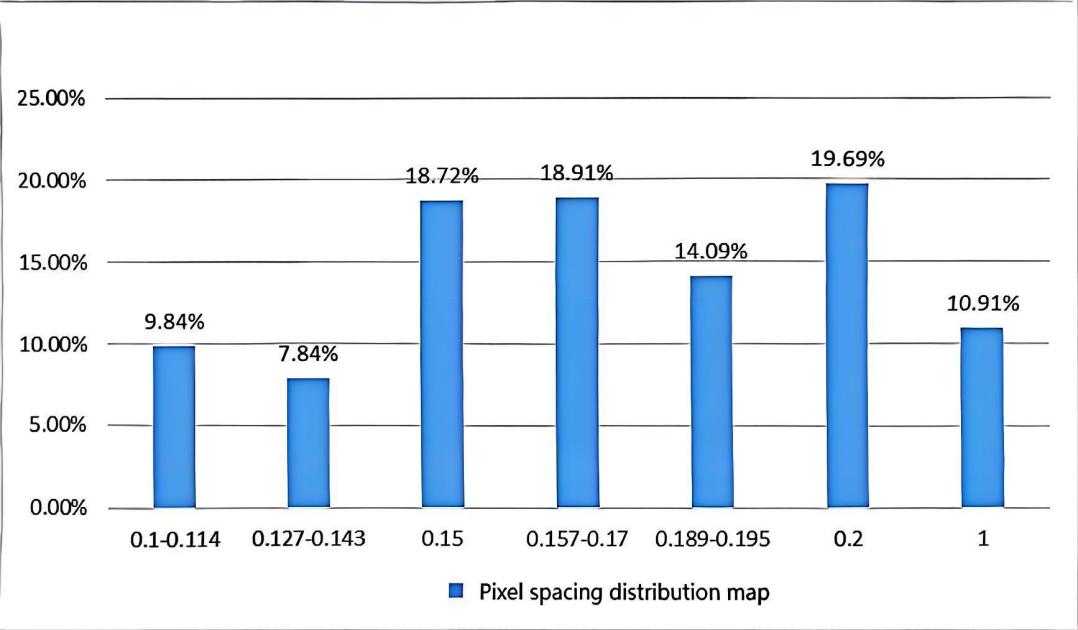


Figure 3. Distribution of pixel spacing of OAI dataset. The data with pixel spacing of 1.0 is the default value due to the missing parameter. Most pixel spacing is less than or equal to 0.2.

Table S1. Distribution of normalized image pixel intensity

| Pixel range | Wide pixel distribution | High pixel distribution |
| --- | --- | --- |
| <1700 | 0.18% | 4.61% |
| 1701-1800 | 0.29% | 74.03% |
| 1801-1900 | 0.67% | 0.58% |
| 1901-2000 | 23.28% | 17.77% |
| 2001-2100 | 2.83% | 2.70% |
| 2101-2240 | 71.81% | 0.30% |
| >2240 | 0.94% | 0.01% |

Reference

1. Singh, A., Horng, H., Roshkovan, L., Weeks, J.K., Hershman, M., 2022.Development of a robust radiomic biomarker of progression-free survival in advanced non-small cell lung cancer patients treated with first-line immunotherapy.Sci Rep. 12(1):9993. doi:10.1038/s41598-022-14160-7.PMID: 35705618
2. Swiecicki, A., Li, N., O'Donnell, J., Said, N., Yang, J.,2021.Deep learning-based algorithm for assessment of knee osteoarthritis severity in radiographs matches performance of radiologists.Comput Biol Med.133:104334.
3. Almhdie-Imjabbar, A., Nguyen, K.L., Toumi, H., Jennane, R., Lespessailles, E.,2022.Prediction of knee osteoarthritis progression using radiological descriptors obtained from bone texture analysis and Siamese neural networks: data from OAI and MOST cohorts.Arthritis Res Ther. 24(1):66. doi: 10.1186/s13075-022-02743-8.PMID: 35260192
4. Tiulpin, A., Thevenot, J., Rahtu, E., Lehenkari, P., Saarakkala, S.,2018.Automatic Knee Osteoarthritis Diagnosis from Plain Radiographs: A Deep Learning-Based Approach.Sci Rep.29;8(1):1727. doi:10.1038/s41598-018-20132-7.PMID: 29379060
