# Supplementary material for: Nomograms Based on X-Ray Radiomics for Predicting Pain Progression in Knee Osteoarthritis Using Data From the Foundation for the National Institutes of Health: Development and Validation Study
Source: JMIR Med Inform. 2026 Jan 14;14:e78338. doi: 10.2196/78338 (PMC12853086; doi:10.2196/78338)
Supplement: Multimedia Appendix 2 [file medinform_v14i1e78338_app2.docx]

Multimedia Appendix 2

Table S1. Baseline characteristics of the subgroup population.

| Characteristic | | | Control group (n=148) | Case group (n=81) | *P* value |
| --- | --- | --- | --- | --- | --- |
| **Demographics** | | | | | |
|  | Age (years), mean (SD) | | 61.81 (8.96) | 59.75 (8.44) | .10 |
|  | Female sex, n (%) | | 94 (63.5) | 51 (63.0) | .93 |
|  | **Race, n (%)** | | | | .18 |
|  |  | Asian | 3 (2.0) | 0 (0.0) |  |
|  |  | Black or African American | 18 (12.2) | 16 (19.8) |  |
|  |  | White | 125 (84.5) | 65 (80.2) |  |
|  |  | Other non-White race | 3 (1.3) | 2 (0.9) |  |
| **Clinical measures** | | | | | |
|  | BMI (kg/m^2^), mean (SD) | | 30.22 (4.92) | 30.85 (5.02) | .32 |
|  | WOMAC^a^ pain score, mean (SD) | | 2.44 (3.09) | 1.70 (2.40) | .09 |
|  | WOMAC function score, mean (SD) | | 7.56 (10.47) | 7.32 (9.19) | .04 |
|  | History of knee injury, n (%) | | 48 (48.0) | 29 (35.80) | .60 |
|  | Use of pain medication, n (%) | | 34 (23.0) | 27 (33.3) | .09 |
| **Radiographic measures** | | | | | |
|  | **Kellgren-Lawrence grade, n (%)** | | | | .85 |
|  |  | 1 | 18 (12.2) | 10 (12.3) |  |
|  |  | 2 | 79 (53.4) | 46 (56.8) |  |
|  |  | 3 | 51 (34.5) | 25 (30.9) |  |
|  | Minimum medial joint space width (mm), mean (SD) | | 3.82 (1.03) | 3.93 (1.08) | .46 |

^a^WOMAC: Western Ontario and McMaster Universities Osteoarthritis Index.

**Table S2. DSCs of automatic segmentation and manual segmentation**

| ROI^a^ | Dice Similarity Coefficients (DSCs) | | |
| --- | --- | --- | --- |
|  | Reader1 (n=40) |  | Reader2 (n=40) |
| ROI 1 | 0.8649±0.0391 |  | 0.8556±0.0378 |
| ROI 2 | 0.9407±0.0108 |  | 0.9390±0.0159 |
| ROI 3 | 0.8848±0.0258 |  | 0.8733±0.0271 |
| ROI 4 | 0.83878±0.0409 |  | 0.8327±0.0410 |

^a^ROI: the region of interest

**Table S3. Sixteen radiomic features of Rad-score1 were extracted using LASSO regression.**

| Filter | classification | features | importance degree |
| --- | --- | --- | --- |
| wavelet-HH | first order | Entropy | -0.006476626 |
| wavelet-HH | gllr | GrayLevelNonUniformityNormalized | 0.014237974 |
| log-sigma-3-0-mm-3D | firstorder | Skewness | 0.024247987 |
| wavelet-LL_glszm | glszm | SmallAreaHighGrayLevelEmphasis | -0.002333187 |
| log-sigma-1-0-mm-3D | glrlm | RunLengthNonUniformityNormalized | -0.018467098 |
| log-sigma-1-0-mm-3D | gldm | DependenceNonUniformityNormalized | -0.046919833 |
| wavelet-LH | firstorder | Mean | 0.003964135 |
| wavelet-HH | gllr | HighGrayLevelRunEmphasis | -0.00206553 |
| wavelet-HH | gldm | HighGrayLevelEmphasis | -0.03827669 |
| wavelet-LL | glrlm | RunVariance | -0.003609233 |
| log-sigma-3-0-mm-3D | firstorder | firstorder_Kurtosis | 0.007154494 |
| log-sigma-3-0-mm-3D | glrlm | LongRunHighGrayLevelEmphasis | -0.016157855 |
| gradient | firstorder | Skewness | 0.006686068 |
| wavelet-HH | glszm | GrayLevelNonUniformityNormalized | -0.003245426 |
| wavelet-LL | glcm | ClusterProminence | -0.001609051 |
| log-sigma-3-0-mm-3D | glrlm | RunEntropy | -0.004368494 |

**Table S4. Twelve radiomic features of Rad-score2 were extracted using LASSO regression.**

| Filter | classification | features | importance degree |
| --- | --- | --- | --- |
| wavelet-HH | first order | Entropy | -0.006720772 |
| wavelet-HH | gllr | GrayLevelNonUniformityNormalized | 0.014352535 |
| log-sigma-1-0-mm-3D | gldm | DependenceNonUniformityNormalized | -0.005118824 |
| log-sigma-3-0-mm-3D | firstorder | kewness | 0.038710816 |
| wavelet-LH | firstorder | Mean | 0.007665185 |
| wavelet-HH | gllr | HighGrayLevelRunEmphasis | -0.011465065 |
| wavelet-HH | gldm | HighGrayLevelEmphasis | -0.040549644 |
| wavelet-LL | LL_glrlm | RunVariance | -0.009958921 |
| log-sigma-1-0-mm-3D | glcm | glcm_SumEntropy | 0.003273765 |
| log-sigma-3-0-mm-3D | firstorder | firstorder_Kurtosis | 0.014244369 |
| log-sigma-3-0-mm-3D | glrlm | LongRunHighGrayLevelEmphasis | -0.018163921 |
| gradient | firstorder | Skewness | 0.008274971 |

**Table S5. Eleven radiomic features of Rad-score3 were extracted using LASSO regression.**

| Filter | classification | features | importance degree |
| --- | --- | --- | --- |
| wavelet-LH | glszm | ZonePercentage | 0.004292576 |
| wavelet-LH | gldm | DependenceVariance | 0.005284267 |
| wavelet-HH | firstorder | Entropy | -0.043856621 |
| wavelet-LL | glszm | SizeZoneNonUniformity | 0.007742425 |
| wavelet-HL | glr lm | GrayLevelNonUniformity | -0.014870537 |
| wavelet-LL | glszm | SmallAreaHighGrayLevelEmphasis | -0.019727717 |
| log-sigma-1-0-mm-3D | gldm | DependenceNonUniformityNormalized | -0.003351462 |
| wavelet-HH | gllr | ShortRunEmphasis | 0.001696458 |
| wavelet-HH | gldm | LargeDependenceHighGrayLevelEmphasis | -0.035630255 |
| wavelet-HL | glr lm | GrayLevelNonUniformityNormalized | 0.001460165 |
| wavelet-LL | firstorder | Kurtosis | -0.004485347 |

| Filter | classification | features | importance degree |
| --- | --- | --- | --- |
| wavelet-HH | first order | Entropy | -0.032555391 |
| wavelet-HH | gllr | ShortRunHighGrayLevelEmphasis | 0.023857409 |
| wavelet-HH | gldm | LargeDependenceHighGrayLevelEmphasis | 0.00367592 |

**Table S6. Three radiomic features of Rad-score4 were extracted using LASSO regression.**


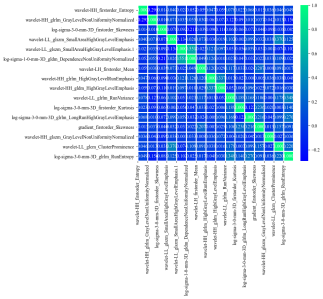

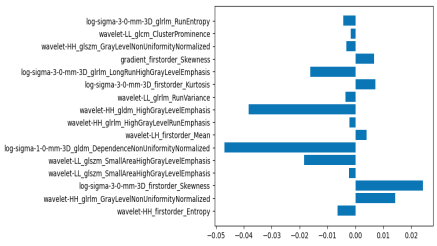

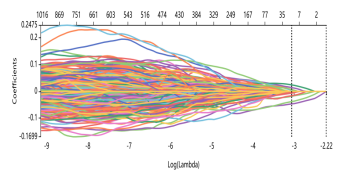

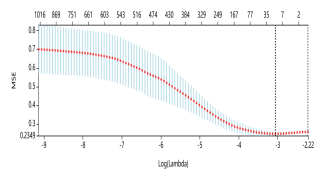


**A**

**D**

**C**

**B**

Figure S1. Feature extraction of Rad-score1. (a) Curve of regression coefcients with Log(λ), which decreases as the coefcients score continues to go down. (b) Non-zero coefficient features. (c) Correlation heat map. (d) Image feature weighted coefficient diagram.


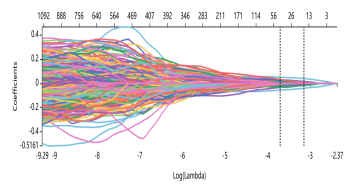

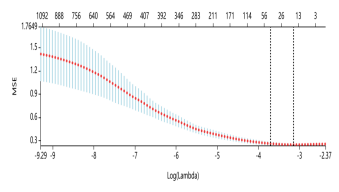

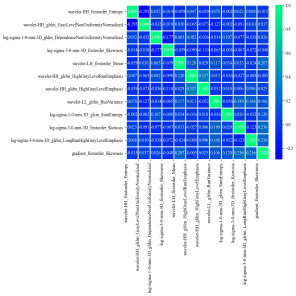

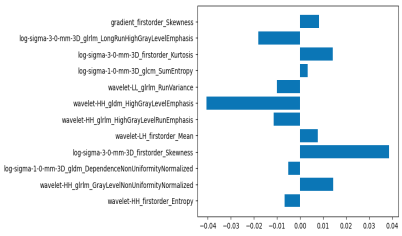


**A**

**D**

**B**

**C**

Figure S2. Feature extraction of Rad-score2. (a) Curve of regression coefcients with Log(λ), which decreases as the coefcients score continues to go down. (b) Non-zero coefficient features. (c) Correlation heat map. (d) Image feature weighted coefficient diagram.


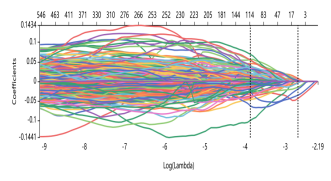

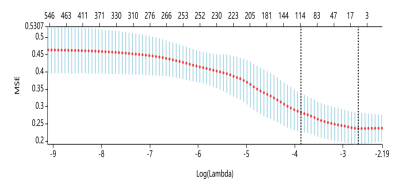

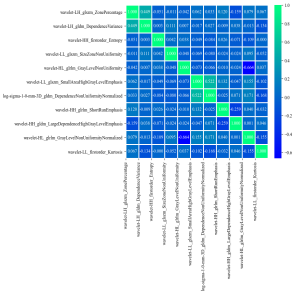

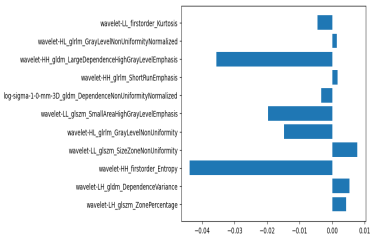


**A**

**D**

**C**

**B**

Figure S3. Feature extraction of Rad-score3. (a) Curve of regression coefcients with Log(λ), which decreases as the coefcients score continues to go down. (b) Non-zero coefficient features. (c) Correlation heat map. (d) Image feature weighted coefficient diagram


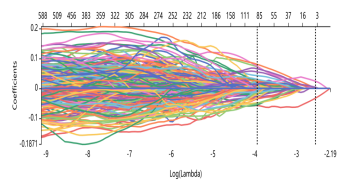

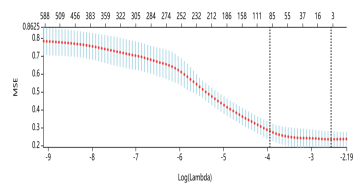

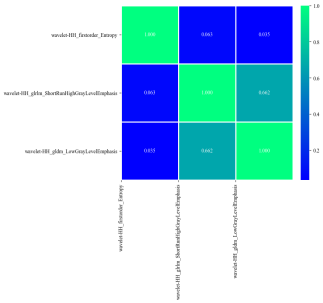

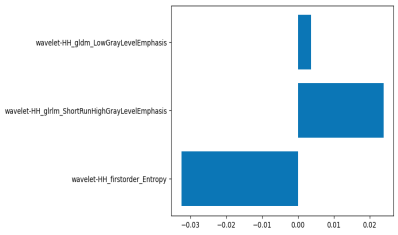


**A**

**B**

**C**

**D**

**D**

Figure S4. Feature extraction of Rad-score4. (a) Curve of regression coefcients with Log(λ), which decreases as the coefcients score continues to go down. (b) Non-zero coefficient features. (c) Correlation heat map. (d) Image feature weighted coefficient diagram


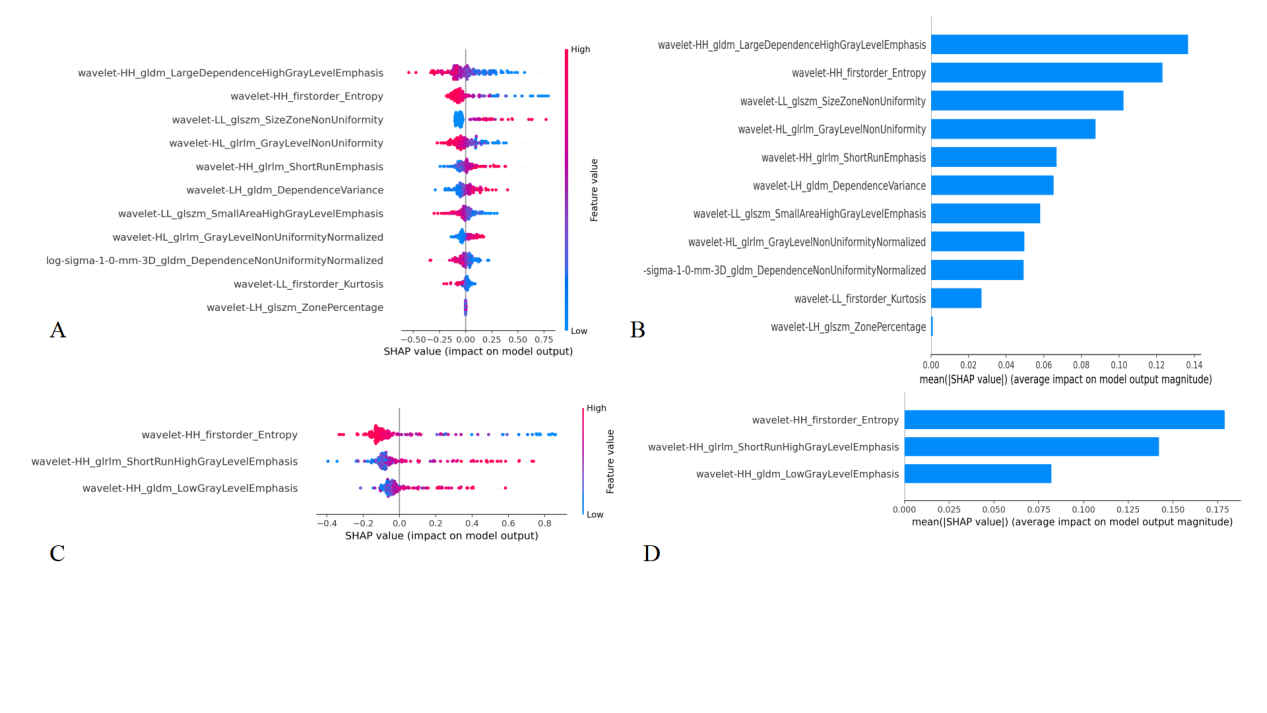


Figure S5. SHAP summary plots of Radiomic Score. The plot illustrated the feature relevance and attributions to the model's predictive performance (A. Rad-Score3; C. Rad-Score 4). The bar chart illustrates the impact of various imaging features on Rad-Score (B. Rad-Score 3; D. Rad-Score 4).
